# Associations between exclusive breastfeeding duration and children’s developmental outcomes: Evidence from Siaya county, Kenya

**DOI:** 10.1371/journal.pone.0265366

**Published:** 2022-03-31

**Authors:** Silas Onyango, Elizabeth Kimani-Murage, Patricia Kitsao-Wekulo, Nelson K. Langat, Kenneth Okelo, Christopher Obong’o, Jürg Utzinger, Günther Fink

**Affiliations:** 1 Swiss Tropical and Public Health Institute, Allschwil, Switzerland; 2 University of Basel, Basel, Switzerland; 3 African Population and Health Research Center, Nairobi, Kenya; 4 PATH, Nairobi, Kenya; Universidade de Sao Paulo Faculdade de Saude Publica, BRAZIL

## Abstract

**Background:**

Exclusive breastfeeding (EBF) during the first 6 months of life is widely promoted as a key strategy to enhance child health, growth, and development. Even though a high proportion of children in Kenya are currently breastfed exclusively, there is little evidence regarding the developmental benefits during the first year of life. This paper aims to fill this gap by establishing an association between EBF and early childhood developmental outcomes among children below the age of 6 months in Kenya.

**Methods:**

We used data collected as part of a cluster-randomized controlled trial conducted in Bondo sub-county in the western part of Kenya to assess the associations between EBF and development in the first year of life. The primary exposure variable was EBF, and the outcome variable was child development as measured by the Ages and Stages Questionnaire–Third Edition (ASQ-3).

**Results:**

We analyzed data from 570 children aged below 6 months at the time of the interview. Breastfeeding children exclusively between 3 and 6 months was associated with 0.61 standard deviation (SD) higher ASQ-3 scores in the adjusted model. When specific domains were considered, in the adjusted models, EBF in the 3–6 months period was associated with 0.44 SD, 0.34 SD and 0.36 SD higher ASQ-3 scores in communication, gross motor, and problem solving domains, respectively. There were weak associations in the fine motor and social-emotional domains.

**Conclusion:**

EBF in the 3- to 6-month age range has significant positive associations with child development, especially for communication, gross motor, and problem-solving. Programs encouraging mothers to continue EBF in this period may have substantial benefits for children.

## Introduction

The period from conception to a child’s third birthday represents the most critical time in the life of a child. This period lays the foundation for the achievement of critical developmental milestones enabling children to reach their full potential [1]. Recent findings indicate that millions of children in low- and middle-income countries (LMICs) fail to achieve their full developmental potential [2]. The majority of these children live in rural or poor urban settings of sub-Saharan Africa [3]. The main causes of poor child development include malnutrition, chronic poverty, and inadequate cognitive and social-emotional stimulation [1, 4]. Understanding children’s growth and development in complex settings within sub-Saharan Africa are important for the design of strategies and interventions that can enhance the achievements of such potentials [4]. Loss of human potential during early childhood periods is associated with later poor schooling and labor outcomes eventually perpetuating the intergenerational cycle of poverty [5].

Exclusive breastfeeding (EBF) for the first 6 months of life, as recommended by the World Health Organization (WHO), is currently considered one of the most effective ways to enhance children’s healthy growth and development [6]. Children who are exclusively breastfed for 6 months have been found to have better nutritional outcomes [7, 8], reduced morbidity [9, 10], and improved early child development [11, 12]. In the long run, breastfeeding has been linked to a lower risk of obesity, asthma, diabetes, and cancer as well as infant mortality [13]. In addition, breastfeeding can enhance mother-child interaction thereby improving bonding between the mother and the child [14]. For mothers, breastfeeding lowers the risks of overweight, postpartum bleeding as well as risks of diabetes and breast cancer [15]. EBF has a strong association with the early achievement of cognitive, social-emotional, language, and fine motor skills [16–20], making it the most effective early intervention program for the children.

Globally, 44% of children are currently estimated to be exclusively breastfed up to 6 months of age [6]. However, EBF rates remain low in many parts of Africa despite major efforts to promote this practice in the past three decades [21]. Kenya remains among the few countries in Africa that have made major progress in EBF rates, with percentages of children breastfed for at least 6 months rising from 32% to 61%, between 2008 and 2014 [22]. The increase in breastfeeding rates is due to intense awareness campaigns on the importance of EBF by the Ministry of Health and early intervention programs such as Baby-Friendly Community Initiative (BFCI) and Baby-Friendly Hospital Initiative (BFHI), currently being implemented in some parts of Kenya [23]. Despite the relatively large benefits of EBF reported in other settings, little is known about the benefits of EBF for at least 6 months and its impact on children’s developmental outcomes in Kenya.

The current paper aims to establish the association between EBF and early childhood developmental outcomes among children aged below 6 months in Kenya. Further, the current paper contributes to the body of literature on the association between EBF and early childhood development among children in sub-Saharan Africa.

## Materials and methods

### Study design

We used data collected as part of a cluster-randomized controlled trial that sought to evaluate the impact of an early childhood development intervention delivered through the health system [24]. Within the study site, 18 public health facilities (clusters) were randomly selected from each of the six wards. The trial comprised a pre-intervention (baseline) survey and a longitudinal follow-up of the caregiver-child dyads for a period of 27 months. This study utilized data collected during the first two follow-ups. The first post-birth follow-up was conducted between September and December 2018 when the children were about 1–2 months, while the second post-birth follow-up was completed between May and August 2019 when the children were about 9–11 months.

### Study site

The study was conducted in Bondo sub-sounty in Siaya county in the western part of Kenya. Siaya county is one of the 47 counties (devolved system of government) formed in 2010 after the promulgation of the current Kenyan constitution. Counties in Kenya are semi-autonomous and are further administrative sub-divided into 6 sub-counties (Bondo, Ugenya, Ugunja, Alego-Usonga, Gem, and Rarieda). Siaya county is situated along the shores of Lake Victoria, where fishing is the main economic activity. As of 2019, the county had a population of 993,183 [25]. Women of reproductive age represent about 23% of the population and the total fertility rate is 4.2, slightly higher than the national rate of 3.9 [26]. About 22.8% of the children in the county below 5 years are stunted (lower than the national rate of 25%) with 12.6% of the children in the same category being underweight compared with the national rate of 11% [27, 28]. With an average household size of 3.8, the Bondo sub-county had a population of 197,883 (95,962 males and 101,917 females) and 20,453 children below the age of 3 years [25] by 2019.

### Study sample

The study sample consisted of 792 pregnant women recruited at baseline (pre-birth) and followed up 570 and 610 primary caregiver-child dyads at post-birth one and post-birth two data collection rounds, respectively. The details of the recruitment process and the design are provided in the baseline report [29]. We used data from the post-birth 1 and post-birth 2 data collection time points. At post-birth 1, the majority of the children were between 1- and 2-month-old with some children extending up to 7 months. At post-birth 2, the majority of the children were between 9 and 11 months old with the youngest child being 4 months and the oldest child being 15 months. Our sample consisted of children below the age of 6 months irrespective of the round of data collection.

### Exposure and outcome variables

The main exposure variable was the practice of EBF as reported by the caregivers during the two rounds of data collection. We defined EBF according to the WHO indicator for infant and young child feeding (IYCF) practices [30], that is, giving only breast milk to the infant (directly from the breast or expressed) and nothing else to drink or eat except for vitamin/mineral supplements or medicines. Non-exclusive breastfeeding was defined as the child having been given other liquids and/or foods other than breast milk. As part of the survey interview, all caregivers were asked “Apart from breast milk, has your child ever been given any food or liquid?” (yes = 1, no = 0). Children were classified as exclusively breastfed (EBF = 1) if the mother reported that they had not given any other foods or liquids and not exclusively breastfed (EBF = 0) if the mother reported that they had ever given the child other foods or liquids. We further categorized the EBF stage as per the age range at which the child was observed, that is, 0–2 months (onset of EBF) and 3–6 months (core EBF period). The primary outcome of interest for the current paper was child development at the age at which the child was observed, between 0–6 months.

Child development was measured using the Ages and Stages Questionnaire–Third Edition (ASQ-3) [31]. The ASQ has been validated and used in Zambia [32] and has also been used in Kenya to assess children’s developmental outcomes [33]. The domains captured in the ASQ-3 include communication, gross motor, fine motor, problem-solving, and personal-social development. The questionnaire was administered by trained field interviewers and relied on caregiver self-reported responses. Six age-specific questions (items) were asked under each domain. Responses were quantified as follows: “yes” (= 10 points) if the child was able to perform the activity; “sometimes” (= 5 points) if the child tried and failed but the caregiver reported that the child could perform the activity; and, “No” (= 0 points) if the child was unable to perform the activity. Responses to all the items in each domain were summed to obtain an aggregate score.

Data were collected at the health facility during the intervention implementation period. All the caregivers were interviewed at the facility as they come to the facility for immunization services. Data were gathered by field interviewers who were trained on conducting interviews with mothers/caregivers. The assessors had at least a bachelor’s degree level of education in child development or related subjects and had experience with the administration of the ASQ tool. We used the ASQ tool to obtain information on child development as part of both follow-up rounds and also inquired about feeding practices in each round.

### Covariate measures

Based on the literature [34–37], we included in our model other covariates that appear to be related to EBF and child development as control variables. The variables included caregiver’s age (in years), occupation, marital status, family size (number of children), family assets (wealth index), and caregiver’s education. Caregiver occupation was conceptualized as either currently employed (including self-employment or small-scale business) or not employed. Marital status was categorized as married or not (divorced, single, or widowed). The number of children consisted of other children the caregiver lived with (including non-biological children), apart from the index child. Caregiver education was categorized as primary education, secondary education, and above secondary education (college and vocational education). The wealth quintile was measured using a household asset scale. Assets such as radio, cellphones, bicycles, motorbikes, television, flush toilet, fridge, and piped water were included in principal component analysis. The predicted value of the first principal component was then used to divide households into wealth quintiles. In addition, we included information on children’s characteristics such as gender, which was obtained during the interview with the mother. All covariates were measured at pre-birth using a structured questionnaire.

### Ethics statement

Ethics approval was obtained from Amref Health Africa’s Ethics and Scientific Review Committee. Permission to conduct the study was first obtained from the National Commission for Science, Technology, and Innovation (NACOSTI) and later from the Siaya County Health Management team (CHMT) before data collection. Written, informed consent was sought and obtained from study participants before any data were collected. For respondents who could neither read nor write, a thumbprint was taken as a signature. Confidentiality of the data and the participants’ privacy were respected at all times. Consenting was done at every round of data collection. Consent documents and the questionnaire were translated into Kiswahili (the national language) and Dholuo (the local language in the study area). The study was registered under trial registration number ISRCTN11561283.

### Statistical analysis

Data management and analysis were performed using STATA version 16.0 for Windows (STATA Corporation, College Station, TX, United States of America) [38]. We presented continuous variables using means and standard deviations (SDs). Categorical variables were summarized as frequencies and percentages. Simple linear regression analyses were performed to estimate the unconditional association between the practice of EBF and ASQ scores overall, as well as during specific sub-periods: the onset period (0–2 months), and the core EBF period (3–6 months). We created an age integer variable for the children observed between 0 and 6 months, irrespective of the data collection round or time point. To examine the association of EBF on ASQ scores conditional on observable characteristics, we used multiple linear regression with heteroscedasticity-robust clustered standard errors (cluster-health facility) controling for the aforementioned covariates. We also controlled for treatment groups and survey round of the larger study in these models.

Further, we determined the association between each of the ASQ domains during the 3- to 6-month age range and EBF using a multiple linear regression with heteroscedasticity-robust clustered standard errors. We presented the results in standardized regression coefficients with the corresponding 95% confidence intervals (Cis) and p-values.

## Results

We analyzed data for 570 children below the age of 6 months at the time of the interview. A total of 270 children were observed at one month, 163 at two months, 76 at three months, 34 at four months, 18 at five months, and 9 at 6 months. In addition, we analyzed 433 children between 0 and 2 months and 137 between 3 and 6 months.

Descriptive statistics of the analytical sample are presented in **Table 1**. In the pooled sample, 86% of the children aged below 6 months were exclusively breastfed. The proportion was higher (88%) for children in the 0-2-month age category with a drop to 73% for children in the 3-6-month age range. About 53% of the children in the EBF group and 42% in the non-EBF group were female. On average, caregivers in the sample were 26 years old, with about two children per family. Most of the caregivers (64.2%) in the EBF group atteined at least primary education. Nearly two-thirds of the caregivers (64.8%) in the EBF group and 73% in the non-EBF group were employed. A high proportion (80%) of the caregivers were married. The ASQ mean score for children who were exclusively breastfed in the 3–6 months age category was 55.1 compared to 51.5 for those not exclusively breastfed in that age category (**Table 2**), these differences were significant (p = 0.004).

**Table 1:** Descriptive statistics (caregivers’ baseline characteristics).

	Exclusively breastfed	Not exclusively breastfed	*p-value*
Child/caregiver’s characteristics	N = 492	N = 78	
Child: female	261 (53.1)	33 (42.3)	0.078
No of children, mean (SD)	2.4 (1.8)	2.4 (1.9)	0.954
0–2 months, n (%)	381 (77.4)	52 (66.7)	0.039
3–6 months, n (%)	111 (73.1)	26 (26.9)	0.039
0–6 months, n (%)	492 (86.3)	78 (13.7)	0.000
Caregiver age, mean (SD)	26.7 (5.9)	25.7 (6.3)	0.162
Primary education, n (%)	318 (64.2)	54 (69.2)	0.428
Secondary education, n (%)	150 (30.5)	19 (24.4)	0.271
Above secondary, n (%)	24 (4.9)	5 (6.4)	0.567
Caregiver employed, n (%)	319 (64.8)	57 (73.1)	0.154
Caregiver married, n (%)	423 (86.0)	62 (79.5)	0.135
Wealth quintile 1	111 (22.6)	14 (18.0)	0.360
Wealth quintile 2	134 (27.4)	24 (30.8)	0.517
Wealth quintile 3	54 (11.0)	15 (19.2)	0.038
Wealth quintile 4	93 (18.9)	15 (19.2)	0.945
Wealth quintile 5	100 (20.3)	10 (12.8)	0.119

**Table 2:** ASQ scores.

	Exclusively breastfed		Not exclusively breastfed		*P-value*
	Mean	SD	Mean	SD	
ASQ-scores 0–2 months	52.0	9.9	52.3	7.5	0.447
ASQ-scores 3–6 months	55.1	6.2	51.5	7.4	0.004
ASQ-scores 0–6 months	52.7	9.3	52.0	7.4	0.829

**Fig 1:**
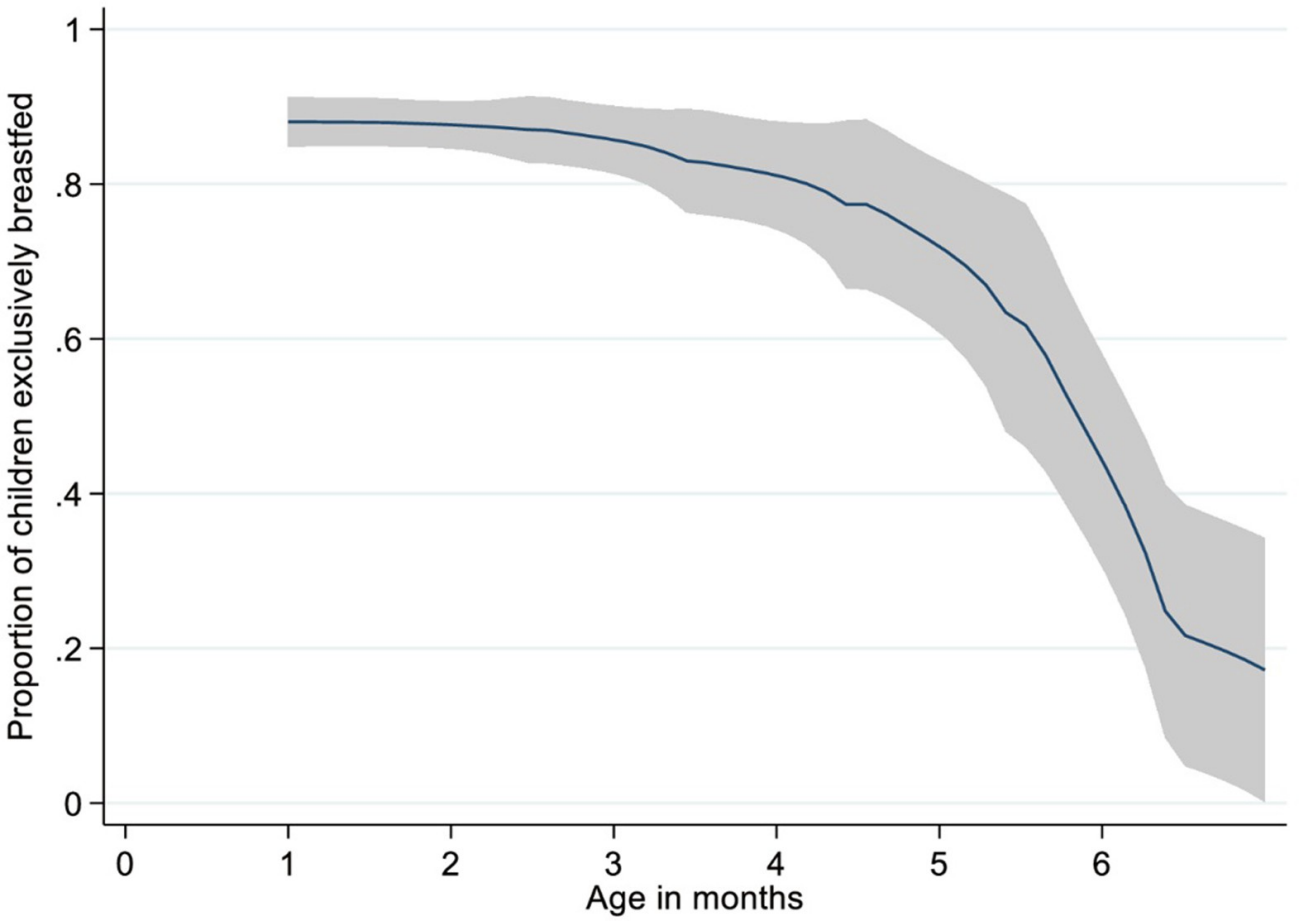
Proportion of exclusive breastfeeding by age in months.

As shown in **Fig 1**, the rates of EBF dropped rapidly from four months. During the first month, higher proportions (>80%) of children were exclusively breastfed. The rates drastically reduced to below 50% during the sixth month.

**Fig 2:**
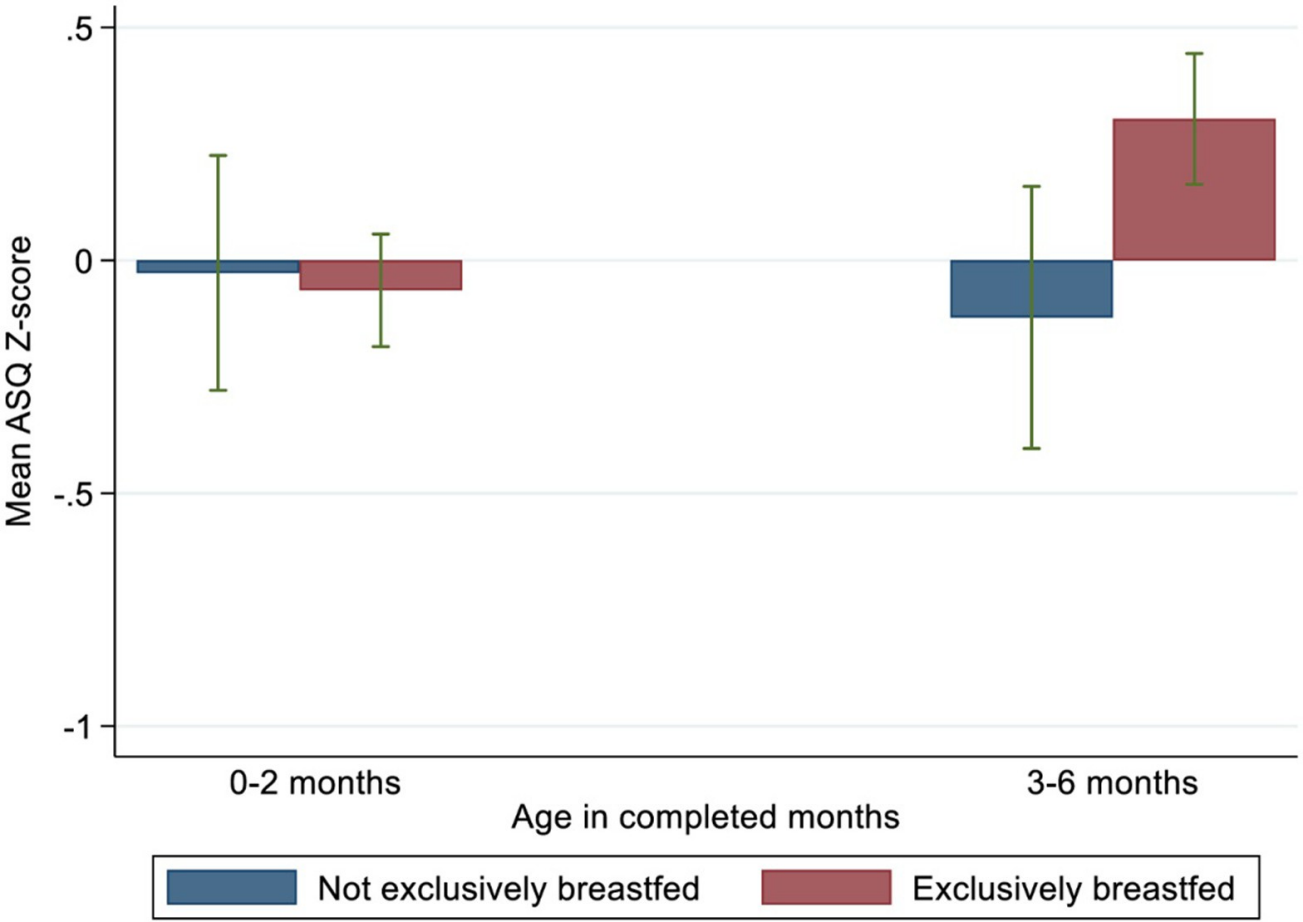
Children’s mean ASQ Z–scores and age groups in months by EBF group.

As shown in **Fig 2**, major differences were observed between the EBF group and no EBF group in the mean developmental outcome scores for the 3-6-month period, where the EBF group had significantly higher mean score (p = 0.004). The differences observed in the early period (0–2 months) were minor.

Information on the crude and the adjusted association between EBF and the children’s ASQ-3 mean scores is presented in **Table 3**. In the unadjusted model, being exclusively breastfed between 3 and 6 months was associated with 0.53 SD higher ASQ mean scores than not being exclusively breastfed (95% CI [0.03–1.03]). After adjusting for covariates, children in the 3-6-months age range who were exclusively breastfed had 0.61 SD higher ASQ-3 mean scores than those who were not (95% CI [0.19–1.03]). Crude and adjusted models did not show any association between ASQ-3 mean scores at 0-2-month age range, and the overall period (0–6 months).

**Table 3:** Unadjusted and adjusted regression for the associations between current EBF and child development (ASQ scores) ages 0–6 months.

	Unadjusted associations			Adjusted associations		
Variable	0–6 months	0–2 months	3–6 months	months	0–2 months	3–6 months
Exclusive breastfeeding	0.11	-0.04	0.53**	0.23	0.19	0.61***
	(-0.23–0.45)	(-0.53–0.45)	(0.03–1.03)	(-0.05–0.52)	(-0.20–0.58)	(0.19–1.03)
Caregiver’s age				0.01	0.00	0.01
				(-0.02–0.03)	(-0.02–0.03)	(-0.02–0.04)
Caregiver employed				-0.07	-0.16	0.24*
				(-0.29–0.15)	(-0.39–0.07)	(-0.01–0.49)
Caregiver married				0.02	0.16	-0.22
				(-0.44–0.48)	(-0.39–0.72)	(-0.54–0.09)
No. of children				-0.04	-0.07	-0.00
				(-0.15–0.08)	(-0.19–0.05)	(-0.10–0.09)
Child: female				0.03	0.13	-0.26**
				(-0.14–0.20)	(-0.08–0.34)	(-0.50–-0.01)
Secondary education				0.02	0.03	-0.03
				(-0.25–0.29)	(-0.28–0.34)	(-0.32–0.26)
Above secondary education				-0.31**	-0.53**	0.03
				(-0.60–-0.01)	(-1.03–-0.03)	(-0.40–0.46)
Wealth quintile 2				0.01	0.00	-0.02
				(-0.22–0.24)	(-0.26–0.26)	(-0.48–0.44)
Wealth quintile 3				0.20	0.16	0.16
				(-0.04–0.44)	(-0.13–0.46)	(-0.34–0.66)
Wealth quintile 4				0.15	0.13	0.05
				(-0.14–0.44)	(-0.17–0.42)	(-0.41–0.51)
Wealth quintile 5				0.29**	0.29**	0.41*
				(0.02–0.56)	(0.01–0.57)	(-0.07–0.89)
Constant	-0.09	-0.03	-0.23	0.03	-0.05	0.06
	(-0.43–0.24)	(-0.41–0.36)	(-0.80–0.35)	(-0.88–0.95)	(-1.10–1.00)	(-0.84–0.97)
Observations	570	433	137	570	433	137

Notes: *** p<0.01,

** p<0.05,

* p<0.1; Each column presents the standardized coefficients of linear regression for the ASQ scores in each age group, with 95% confidence intervals. In the adjusted associations, the models were also adjusted for the study arm and the survey round.

We presented adjusted associations between the practice of EBF during the 3-6-month period and all the ASQ domains separately (**Table 4**). The estimated associations between EBF in the 3-6-month period and ASQ scores were 0.44 SD for communication (95% CI [0.08–0.79]), 0.34 SD for gross motor (95% CI [0.01–0.66]), and 0.36 SD problem solving (95% CI [0.05–0.66]) with small differences in fine motor and personal social skills.

**Table 4:** Adjusted association between EBF and early childhood development (ECD) domains at 3–6 months (core EBF period).

Variable	Communication	Gross motor	Fine motor	Problem-solving	Personal-social
Exclusive breastfeeding	0.44**	0.34**	0.40*	0.36**	0.58*
	(0.08–0.79)	(0.01–0.66)	(-0.02–0.82)	(0.05–0.66)	(-0.05–1.21)
Observations	137	137	137	137	137

Notes: ***p<0.01,

**p<0.05,

*p<0.1; Each column presents the standardized coefficients of linear regression model for each of the ASQ domain scores, with 95% confidence intervals. The models were adjusted for the covariates in **Tables 1 and 2** including the study arm and survey round. These included caregiver age, caregiver employed, number of children, child’s age at assessment, gender, caregivers’ education, marital status, household assets.

## Discussion

We compared children who were exclusively breastfed to those who were not across different ages in the first 6 months of life. Our results show that large percentages of infants were reported to be exclusively breastfed in the first 3 months of life, with rapid declines in the subsequent months. Our results suggest that infants who were exclusively breastfed between the 3-6-month period showed better development compared to those who were not. In addition, we found that infants who were exclusively breastfed during the core EBF period had higher scores in communication, gross motor, and problem-solving compared to their non-EBF counterparts. Our findings that 88% were exclusively breastfed during the first 2 months and over 70% between ages 3 and 6 months reflect the efforts by the Ministry of Health that have included key early intervention programs such as nurturing care and BFCI. High proportions of EBF during the first 2 months of breastfeeding are expected since most children are still taken for immunization visits. The cessation of EBF after 2 months has been linked to returning to work/businesses, perceived low milk quantity, and negative perception about EBF [39–42].

Previous studies that looked at the associations between breastfeeding and child development found that short periods (0–2 months) of breastfeeding did not show any significant differences between children exclusively breastfed and those who were not since the percentage differences were minimal [43]. These existing findings compare to the current study that has shown no association during the first 2 months of breastfeeding; mainly because most children are exclusively breastfed during the first 2 months of EBF. The Kenyan government supports breastfeeding mothers during the first three months of the child’s life by provision of paid leaves for those working [44]. During this period, more than 85% of children in our sample were exclusively breastfed, and hence, no differences were observed in terms of developmental outcomes. Associations between EBF and general child development are documented in the literature [11, 12, 45–47]. While these studies did not use ASQ-3 and were conducted in both low- and high-income settings, the age of assessment for the developmental outcomes ranged from birth to first child’s birthday.

Our findings at 3–6 months, which indicated that EBF was associated with child development, corroborate with existing literature that has found large benefits of EBF on infant development at 4 months [11]. On the other hand, our results indicate that children who are not exclusively breastfed may display poor development, poor health, and nutritional outcomes and may not achieve their full developmental potentials. Our findings on poor development for children not exclusively breastfed corroborate those from a study conducted by Khan and Islam in 2017 [7] on Bangladeshi children that found that a lack of EBF up to 6 months had negative consequences on the health such as frequent diarrhea or fever and nutritional outcomes such stunting or underweight. Also, EBF has been found to have a protective effect against pertussis-like illnesses such as diphtheria or tetanus for children exclusively breastfed for at least 6 months [48]. The association between EBF and language development that was evidenced in our study extends the results of previous studies, which showed profound effects on the early acquisition, and development of receptive and expressive language at 6 months [16, 49]. Our findings that EBF is positively linked to socioemotional development are consistent with prior studies [11, 20] that found a positive effect of breastfeeding on social development for both infants and older children. These findings relate to previous studies [11, 12] that correlated EBF with achievements of cognitive milestones in infants [50]. Early acquisition of language, cognitive skills have a sustained impact on the later academic achievement with an enhanced enrollment of the children [51, 52] and more importantly, these domains are useful indicators of overall development during the early years. Our study also suggests a link between EBF and gross motor skills for children younger than 6 months, which has not been found in other recent studies [53]. While we did not find any sustained effect of exclusive breastfeeding on socioemotional development and fine motor skills, previous studies have documented these relationships, but for children older than 6 months [11, 20].

There are some limitations worth noting. First, child development measures relied on the primary caregiver’s self-report, which may raise the possibilities of social desirability bias among the caregivers. Secondly, children were classified as exclusively breastfed if mothers reported current breastfeeding as well as never having given the child any food or liquids. The responses were dependent on whether the caregivers were able to remember these activities. In addition, the definition of EBF differs from the conventional definition that uses 24-hour recall, and again these may raise the possibility of recall biases.

## Conclusion

The results presented in this paper indicate a relatively strong association between EBF and child development in the 3-6-month age range, especially for communication, gross motor, and problem-solving domains. A key policy priority should therefore focus on the promotion of EBF in this period where many mothers stop EBF in practice.
